# Supplementary material for: Experiences and perceptions of patients with ankylosing spondylitis: A systematic review and meta-synthesis of qualitative studies
Source: PLoS One. 2024 Oct 17;19(10):e0311798. doi: 10.1371/journal.pone.0311798 (PMC11486380; doi:10.1371/journal.pone.0311798)
Supplement: S1 Appendix — (DOCX) [file pone.0311798.s001.docx]

**PubMed**

#1 Spondylitis, Ankylosing [Mesh] OR Ankylosing Spondylitis [Title/Abstract] OR radiographic axial spondyloarthritis [Title/Abstract].

#2 experience [Title/Abstract] OR feeling [Title/Abstract] OR perception [Title/Abstract] OR attitude [Title/Abstract] OR needs [Title/Abstract] OR expectations [Title/Abstract] OR views [Title/Abstract] OR opinion [Title/Abstract] OR belief [Title/Abstract].

#3 qualitative research [Title/Abstract] OR qualitative study [Title/Abstract] OR qualitative methods [Title/Abstract] OR qualitative research [Title/Abstract] OR phenomenology [Title/Abstract] OR grounded theory [Title/Abstract] OR narrative [Title/Abstract] OR ethnography [Title/Abstract] OR interview [Title/Abstract].

#4 #1 AND #2 AND #3

**Web of science**

#1 TS=Spondylitis, Ankylosing OR Ankylosing Spondylitis OR radiographic axial spondyloarthritis.

#2 TS=experience OR feeling OR perception OR attitude OR needs OR expectations OR views OR opinion OR belief.

#3 TS=qualitative research OR qualitative study OR qualitative methods OR qualitative research OR phenomenology OR grounded theory OR narrative OR ethnography OR interview.

#4 #1 AND #2 AND #3

**Embase**

#1. 'spondylitis, ankylosing':ti,ab,kw OR 'ankylosing spondylitis':ti,ab,kw OR 'radiographic axial

spondyloarthritis':ti,ab,kw

#2. experience:ti,ab,kw OR feeling:ti,ab,kw OR perception:ti,ab,kw OR attitude:ti,ab,kw OR needs:ti,ab,kw OR expectations:ti,ab,kw OR views:ti,ab,kw OR opinion:ti,ab,kw OR belief:ti,ab,kw

#3. 'qualitative methods':ti,ab,kw OR 'qualitative research':ti,ab,kw OR phenomenology:ti,ab,kw OR 'grounded theory':ti,ab,kw OR narrative:ti,ab,kw OR ethnography:ti,ab,kw OR interview:ti,ab,kw

#4. #1 AND #2 AND #3

**Cochrane Library**

#1 (Spondylitis, Ankylosing OR Ankylosing Spondylitis OR radiographic axial spondyloarthritis):ti,ab,kw

#2 (experience OR feeling OR perception OR attitude OR needs OR expectations OR views OR opinion OR belief): ti,ab,kw

#3 (qualitative research OR qualitative study OR qualitative methods OR qualitative research OR phenomenology OR grounded theory OR narrative OR ethnography OR interview):ti,ab,kw

#4 #1 AND #2 AND #3

**CINAHL**

SU (Spondylitis, Ankylosing OR Ankylosing Spondylitis OR radiographic axial spondyloarthritis) AND SU (experience OR feeling OR perception OR attitude OR needs OR expectations OR views OR opinion OR belief) AND SU (qualitative research OR qualitative study OR qualitative methods OR qualitative research OR phenomenology OR grounded theory OR narrative OR ethnography OR interview)

**Scopus**

#1 (Spondylitis, Ankylosing OR Ankylosing Spondylitis OR radiographic axial spondyloarthritis):ti,ab,kw

#2 (experience OR feeling OR perception OR attitude OR needs OR expectations OR views OR opinion OR belief): ti,ab,kw

#3 (qualitative research OR qualitative study OR qualitative methods OR qualitative research OR phenomenology OR grounded theory OR narrative OR ethnography OR interview):ti,ab,kw

#4 #1 AND #2 AND #3

**CNKI**

SU=强直性脊柱炎+放射学阳性的中轴型脊柱关节炎+强直性脊柱关节炎+强直性脊椎关节炎ANDSU=经验+体验+感受+经历+需求+期望+态度+感知+观点+信念ANDSU定性+质性研究+扎根理论+现象学+民族志+人种学+访谈

**Wanfang**

主题:(“强直性脊柱炎”+“放射学阳性的中轴型脊柱关节炎”+“强直性脊柱关节炎”+“强直性脊椎关节炎”) AND主题:(“经验”+“体验”+“感受”+“经历”+“需求”+“期望”+“态度”+“感知”+“观点”+“信念”) AND主题:(“定性”+“质性研究”+“扎根理论”+“现象学”+“民族志”+“人种学”+“访谈”)

**CBM**

Abstract:((强直性脊柱炎 OR 放射学阳性的中轴型脊柱关节炎 OR 强直性脊柱关节炎 OR 强直性脊椎关节炎) AND (经验 OR 体验 OR 感受 OR 经历 OR 需求 OR 期望 OR 态度 OR 感知 OR 观点 OR 信念) AND (定性 OR 质性研究 OR 扎根理论 OR 现象学 OR 民族志 OR 人种学 OR 访谈))

**VIP**

(M=(强直性脊柱炎+放射学阳性的中轴型脊柱关节炎+强直性脊柱关节炎+强直性脊椎关节炎) OR R=(强直性脊柱炎+放射学阳性的中轴型脊柱关节炎+强直性脊柱关节炎+强直性脊椎关节炎)) AND (M=(经验+体验+感受+经历+需求+期望+态度+感知+观点+信念) OR R=(经验+体验+感受+经历+需求+期望+态度+感知+观点+信念)) AND (M=(定性+质性研究+扎根理论+现象学+民族志+人种学+访谈) OR R=(定性+质性研究+扎根理论+现象学+民族志+人种学+访谈))
