# Supplementary material for: Experiences and perceptions of patients with ankylosing spondylitis: A systematic review and meta-synthesis of qualitative studies
Source: PLoS One. 2024 Oct 17;19(10):e0311798. doi: 10.1371/journal.pone.0311798 (PMC11486380; doi:10.1371/journal.pone.0311798)
Supplement: S1 Table — (DOCX) [file pone.0311798.s003.docx]

List of included 11 studies.

| **Number** | **Title** |
| --- | --- |
| 1 | Living with pain in ankylosing spondylitis: a qualitative study. |
| 2 | A Novel Qualitative Study Assessing Patient-Reported Outcome Measures Among People Living with Psoriatic Arthritis or Ankylosing Spondylitis. |
| 3 | Patient perspectives of managing fatigue in Ankylosing Spondylitis, and views on potential interventions: a qualitative study. |
| 4 | Men's experiences of living with ankylosing spondylitis. A qualitative study. |
| 5 | Fatigue in ankylosing spondylitis: causes, consequences and self-management. |
| 6 | Living With a Fluctuating Illness of Ankylosing Spondylitis: A Qualitative Study. |
| 7 | Life Strain-Related Tiredness and Illness-Related Fatigue in Individuals With Ankylosing Spondylitis. |
| 8 | The new perceptions on life of Iranian patients with ankylosing spondylitis: A qualitative study. |
| 9 | A Difficult Diagnosis: A Qualitative Study of the Daily Lives of Young Men Diagnosed with Ankylosing Spondylitis. |
| 10 | 中青年强直性脊柱炎病人疾病感受和体验的质性研究 |
| 11 | 青年强直性脊柱炎患者不同阶段疾病感知特征的质性研究 |

List of included 577 studies.

| **Number** | **Title** |  |
| --- | --- | --- |
| Reason for exclusion: Irrelevant title or abstract. (n=541) | |  |
| 1 | Prevalence of Axial Spondyloarthritis Among Patients With Fibromyalgia: A Magnetic Resonance Imaging Study With Application of the Assessment of SpondyloArthritis International Society Classification Criteria |  |
| 2 | Direct and indirect costs associated with ankylosing spondylitis and related disease activity scores in Turkey |  |
| 3 | Google Internet Searches Related to Inflammatory Arthritis: an Observational Study Using Google Trends Data |  |
| 4 | The Impact of Spondyloarthritis on Health-Related Quality of Life and Healthcare Resource Utilization in Saudi Arabia: A Narrative Review and Directions for Future Research |  |
| 5 | COVID-19 Mortality in Patients with Rheumatic Diseases: A Real Concern |  |
| 6 | Cladribine Tablets and Relapsing–Remitting Multiple Sclerosis: a Pragmatic, Narrative Review of What Physicians Need to Know |  |
| 7 | Information needs of patients with spondyloarthritis about their disease |  |
| 8 | Vaccination status of patients using anti-TNF therapy and the physicians' behavior shaping the phenomenon: Mixed-methods approach |  |
| 9 | Medication adherence and persistence in patients with autoimmune rheumatic diseases: a narrative review |  |
| 10 | Cardiovascular risk in ankylosing spondylitis and the effect of anti-TNF drugs: a narrative review |  |
| 11 | Experiences of mothers living with inflammatory arthritis |  |
| 12 | Data to be collected for an optimal management of axial spondyloarthritis in daily practice: Proposal from an evidence based and consensual approaches |  |
| 13 | Feasibility of utility assessment by rating scale and standard gamble in patients with ankylosing spondylitis or fibromyalgia |  |
| 14 | Effectiveness and cost-effectiveness of a multimodal, physiotherapist-led, vocational intervention in people with inflammatory arthritis: study protocol of the Physiotherapy WORKs trial |  |
| 15 | Immunogenicity of anti-TNF therapies in patients with inflammatory rheumatic diseases and secondary failure: a multicentre study of 570 patients |  |
| 16 | What constitutes the fat signal detected by MRI in the spine of patients with ankylosing spondylitis? A prospective study based on biopsies obtained during planned spinal osteotomy to correct hyperkyphosis or spinal stenosis |  |
| 17 | Vivências das pessoas idosas com doença reumática inflamatória sistémica a realizar terapêutica biológica na consulta de enfermagem |  |
| 18 | Work disability among people with ankylosing spondylitis |  |
| 19 | Sentido de Vida da Pessoa com dor Crónica |  |
| 20 | Unmet Information Needs of Patients with Rheumatic Diseases: Results of a Cross-Sectional Online Survey Study in Germany |  |
| 21 | Development of a web-based decision aid for initiating biological or targeted synthetic disease-modifying antirheumatic drugs (b/tsDMARDs) in axial spondyloarthritis |  |
| 22 | Validation and implementation of a patient-reported experience measure for patients with rheumatoid arthritis and spondyloarthritis in the Netherlands |  |
| 23 | Non-radiographic axial spondyloarthritis in Tunisia: main characteristics and detailed comparison with ankylosing spondylitis |  |
| 24 | Structured Goal Planning and Supportive Telephone Follow-up in Rheumatology Care: results From a Pragmatic, Stepped-Wedge, Cluster-Randomized Trial |  |
| 25 | Fears and Beliefs in Rheumatoid Arthritis and Spondyloarthritis: A Qualitative Study |  |
| 26 | Uveitis is associated with hypertension and atherosclerosis in patients with ankylosing spondylitis: A cross-sectional study |  |
| 27 | A qualitative exploration of the personal financial toll of arthritis |  |
| 28 | How Do Patients With Axial Spondyloarthritis Experience High-Intensity Exercise? |  |
| 29 | Sex and Gender Interactions in the Lives of Patients with Spondyloarthritis in Spain: A Quantitative-qualitative Study |  |
| 30 | Patient experience with intravenous biologic therapies for ankylosing spondylitis, Crohn's disease, psoriatic arthritis, psoriasis, rheumatoid arthritis, and ulcerative colitis |  |
| 31 | A comparison of patients with spondyloarthropathy seen in specialty clinics with those identified in a communitywide epidemiologic study. Has the classic case misled us? |  |
| 32 | Evaluation of the European Spondylarthropathy Study Group preliminary classification criteria in Alaskan Eskimo populations |  |
| 33 | Emerging therapies for the treatment of spondyloarthritides with focus on axial spondyloarthritis |  |
| 34 | The role of HLA-B27 in the pathogenesis and diagnosis of axial spondyloarthritis: 50 years after discovery of the strong genetic association |  |
| 35 | Physical Activity and Health Perception in Inflammatory Joint Disease : a Physiotherapy Perspective |  |
| 36 | It's Like Listening to the Radio with a Little Interference: A Qualitative Study Describing Pain Management among Patients with Psoriatic Arthritis |  |
| 37 | Important problems identified by patients with chronic arthritis |  |
| 38 | Prevalence and characteristics of hip involvement in spondyloarthritis: a single-centre observational study of 275 patients |  |
| 39 | Prevalence of complementary and alternative medicine despite limited perceived efficacy in patients with rheumatic diseases in Mexico: Cross-sectional study |  |
| 40 | Management of patients with inflammatory rheumatic diseases after treatment failure with a first tumour necrosis factor inhibitor: A narrative review |  |
| 41 | Patients' perception on disease progression and adherence to biologic therapy |  |
| 42 | Equivalence of paper and electronic administration of patient reported outcomes: a comparison in psoriatic arthritis |  |
| 43 | Opportunities and challenges for real-world studies on chronic inflammatory joint diseases through data enrichment and collaboration between national registers: the Nordic example |  |
| 44 | Ankylosing spondylitis & chronic pain syndrome: Bridging the gap between perpetuated medicine & holistic therapies |  |
| 45 | Osteoarthritis: new roles for drug therapy and surgery. Interview by Peter Pompei |  |
| 46 | Genetic markers as a predictive tool based on statistics in medical practice: ethical considerations through the analysis of the use of HLA-B*27 in rheumatology in France |  |
| 47 | Benefits of Crenotherapy in the Treatment of Rheumatic Diseases |  |
| 48 | Attitudes of patients with a rheumatic disease on drug use in the COVID-19 pandemic |  |
| 49 | Impact of Routines and Rituals on Burden of Treatment, Patient Training, Cognitive Load, and Anxiety in Self-Injected Biologic Therapy |  |
| 50 | Use of conventional and unconventional therapies among patients with ankylosing spondylitis |  |
| 51 | The use of focus group interview in the evaluation of patients with ankylosing spondylitis |  |
| 52 | Digital Additional Risk Minimization Measures: An Exploratory Study Using Qualitative Feedback from Healthcare Professionals and Patients Across Six Countries |  |
| 53 | Safety of treatment options for spondyloarthritis: a narrative review |  |
| 54 | The prevalence of musculoskeletal complaints in a rural area in Iran: a WHO-ILAR COPCORD study (stage 1, rural study) in Iran |  |
| 55 | Effect of ethnic origin (Caucasians versus Turks) on the prevalence of rheumatic diseases: a WHO-ILAR COPCORD urban study in Iran |  |
| 56 | A multidisciplinary job retention vocational rehabilitation programme for patients with chronic rheumatic diseases: patients' and occupational physicians' satisfaction |  |
| 57 | Content analysis of Twitter in relation to biological treatments for chronic inflammatory arthropathies: An exploratory study |  |
| 58 | Current Evidence of the Management of Undifferentiated Spondyloarthritis: A Systematic Literature Review |  |
| 59 | What Has Been the Effect on Trial Outcome Assessments of a Decade of Patient Participation in OMERACT? |  |
| 60 | Prevalence of rheumatic diseases in Raramuri people in Chihuahua, Mexico: a community-based study |  |
| 61 | Restless legs syndrome is associated with poor sleep quality and quality of life in patients with ankylosing spondylitis: a questionnaire-based study |  |
| 62 | Therapeutic drug monitoring of biologicals in rheumatoid arthritis: a disconnect between beliefs and facts |  |
| 63 | Development of the ASQoL: a quality of life instrument specific to ankylosing spondylitis |  |
| 64 | Personal Experiences with Diagnostic Delay Among Axial Spondyloarthritis Patients: A Qualitative Study |  |
| 65 | Fatigue in inflammatory arthritis |  |
| 66 | Translation and validation of the Turkish version of the Ankylosing Spondylitis Quality of Life (ASQOL) questionnaire |  |
| 67 | Similarities and differences in the experience of fatigue among people living with fibromyalgia, multiple sclerosis, ankylosing spondylitis and stroke |  |
| 68 | The arthritic patients' perspective of measuring treatment efficacy: Patient Reported Experience Measures (PREMs) as a quality tool |  |
| 69 | Sexual problems in rheumatoid arthritis and ankylosing spondylitis |  |
| 70 | Women of steel and bamboo: Narratives of meaning -making in women with ankylosing spondylitis |  |
| 71 | Can-Art Effect and safety of using Canabis derivatives for the treatment of pain in patients with inflammatory Arthritis, such as reumatoid arthritis and ankylosing spondylitis, the latter being a type of arthritis that causes a long term inflammation of the joints of the spine. A randomized, double blinded, placebo controlled trial, i.e. in this drug trial, a control group is given a placebo while another group is given the Cannabis derivative being studied |  |
| 72 | A phase IV, randomized, open-label, controlled, post-licensure study to evaluate the safety of GlaxoSmithKline Biologicals’ HPV-16/18 L1 VLP AS04 vaccine (Cervarix®) when administered intramuscularly according to a 0, 1, 6-month schedule in females aged 18-25 years. - EPI-HPV-111103 |  |
| 73 | Symptoms, effects on quality of life, judgement and expectations of treatment in active ankylosing spondylitis: the patient's view |  |
| 74 | Retained and discontinued conventional and unconventional measures by patients with Bechterew disease for modifying the course of illness |  |
| 75 | A truly complementary approach: A qualitative exploration of complementary and alternative medicine practitioners' views of treating ankylosing spondylitis |  |
| 76 | Do physical therapists follow evidence-based practices for treatment of inflammatory arthritis? Results from an online survey |  |
| 77 | Clinical management of rheumatologic conditions co-occurring with inflammatory bowel diseases |  |
| 78 | Assessing quality of life of self-reported rheumatic patients |  |
| 79 | Qualitative interviews of symptoms, impacts and selected promis short forms: a study in patients with axial spondyloarthritis |  |
| 80 | Qualitative concept elicitation and cognitive debriefing interviews of symptoms, impacts and selected customized PROMIS Short Forms: a study in patients with axial spondyloarthritis |  |
| 81 | Barriers and Facilitators for Being Physically Active in Patients with Ankylosing Spondylitis: A Cross-sectional Comparative Study |  |
| 82 | Prevalence and clinical characteristics of ankylosing spondylitis in Iceland - a nationwide study |  |
| 83 | Initial Diagnosis of Lumbar Disc Herniation Is Associated with a Delay in Diagnosis of Ankylosing Spondylitis |  |
| 84 | Mobile Health Features Supporting Self-Management Behavior in Patients With Chronic Arthritis: Mixed-Methods Approach on Patient Preferences |  |
| 85 | Beyond dyadic communication: Network of communication in inflammatory arthritis teams |  |
| 86 | Reducing work disability in Ankylosing Spondylitis - development of a work instability scale for AS |  |
| 87 | Development and psychometric validation of a patient-reported outcome measure to assess fears in rheumatoid arthritis and axial spondyloarthritis: the Fear Assessment in Inflammatory Rheumatic diseases (FAIR) questionnaire |  |
| 88 | The role of an anterior approach in the treatment of ankylosing spondylitis-associated cervical fractures: a systematic review and meta-analysis |  |
| 89 | Being a parent or grandparent with back pain, ankylosing spondylitis or rheumatoid arthritis: a descriptive postal survey |  |
| 90 | Exploring IL-17 in spondyloarthritis for development of novel treatments and biomarkers |  |
| 91 | Coping with arthritis is experienced as a dynamic balancing process. A qualitative study |  |
| 92 | Educational needs in patients with spondyloarthritis in Sweden - a mixed-methods study |  |
| 93 | The content validity and acceptability of the Evaluation of Daily Activity Questionnaire in musculoskeletal conditions |  |
| 94 | Psychometric testing of the British-English Long-Term Conditions Job Strain Scale, Long-Term Conditions Work Spillover Scale and Work-Health-Personal Life Perceptions Scale in four rheumatic and musculoskeletal conditions |  |
| 95 | Psychometric testing of the British English Workplace Activity Limitations Scale in four rheumatic and musculoskeletal conditions |  |
| 96 | Patients' experiences with goal pursuit after discharge from rheumatology rehabilitation: A qualitative study |  |
| 97 | I am so much more than my work: a qualitative study of experiences after participating in return to work programs for people with rheumatic diseases |  |
| 98 | Young people's decisions about biologic therapies: who influences them and how? |  |
| 99 | Development of classification criteria for hand osteoarthritis: comparative analyses of persons with and without hand osteoarthritis |  |
| 100 | How do patients with rheumatic disease experience their relationship with their doctors? A qualitative study of experiences of stress and support in the doctor-patient relationship |  |
| 101 | Incorporating FRAX into a nurse-delivered integrated care review: a multi-method qualitative study |  |
| 102 | Barriers and facilitators in diagnosing axial spondyloarthritis: a qualitative study |  |
| 103 | Diagnostic delay in axial spondyloarthritis: a systematic review |  |
| 104 | Optimising daily diary questionnaires about fatigue, psychological flexibility and well-being: perspectives of people with rheumatic disease |  |
| 105 | Patients' Perspectives on Information and Communication About Sexual and Relational Issues in Rheumatology Health Care |  |
| 106 | Rheumatic Diseases and Sexuality: Disease Impact and Self-Management Strategies |  |
| 107 | Estimates of the prevalence of arthritis and other rheumatic conditions in the United States |  |
| 108 | Comparison of two ELISA versions for infliximab serum levels in patients diagnosed with ankylosing spondylitis |  |
| 109 | Usage problems and social barriers faced by persons with a wheelchair and other aids. Qualitative study from the ergonomics perspective in persons disabled by rheumatoid arthritis and other conditions |  |
| 110 | Do Transsacral-transiliac Screws Across Uninjured Sacroiliac Joints Affect Pain and Functional Outcomes in Trauma Patients? |  |
| 111 | Does a regular exercise program for ankylosing spondylitis influence body image? |  |
| 112 | How to optimize exercise behavior in axial spondyloarthritis? Results of an intervention mapping study |  |
| 113 | Hpr pilot implementation of enhancements in supervised group exercise for people with axial spondyloarthritis (axspa) in the netherlands |  |
| 114 | Pilot implementation of enhancements in supervised group exercise for people with axial spondyloarthritis (AXSPA) in the netherlands |  |
| 115 | Working with axspa: A qualitative study using the british society for rheumatology biologics register |  |
| 116 | Outcomes and treatment responses, including work productivity, among people with axial spondyloarthritis living in urban and rural areas: a mixed-methods study within a national register |  |
| 117 | Pain Experiences and Decision-making Needs for Pain Management Among Young Women with Hypermobile Ehlers-Danlos Syndrome and Generalized Joint Hypermobility Spectrum Disorder |  |
| 118 | A comparison of three methods to generate a conceptual understanding of a disease based on the patients' perspective |  |
| 119 | Subcutaneously-Administered Infliximab in the Management of Rheumatoid Arthritis: A Short Narrative Review of Current Clinical Evidence |  |
| 120 | Integrating and improving care for patients with inflammatory rheumatological disorders in the community |  |
| 121 | Trial of stopping or continuing biologics ahead of orthopaedic surgery |  |
| 122 | Patients' utilities for cancer treatments: a study of the chained procedure for the standard gamble and time tradeoff |  |
| 123 | Development of Image Overlay and Knowledge Transfer Module Technologies Aimed at Enhancing Feasibility and External Validation of Magnetic Resonance Imaging-based Scoring Systems |  |
| 124 | Use of Complementary and Alternative Medicine in Axial Spondyloarthritis: A Qualitative Exploration of Self-Management |  |
| 125 | Effects of additional context information in prescription drug information sheets on comprehension and risk and efficacy perceptions |  |
| 126 | A lower frequency of inflammatory back pain in male patients with ankylosing spondylitis compared with female patients |  |
| 127 | The disease burden of axial spondyloarthritis: through a gendered lens |  |
| 128 | The role of enjoyment in exercise for people with arthritis: Four different viewpoints from a Q-methodology study |  |
| 129 | A mixed methods study to uncover impediments to accurate diagnosis of nonradiographic axial spondyloarthritis in the USA |  |
| 130 | Narrative Review of the Mechanism of Hip Prosthesis Dislocation and Methods to Reduce the Risk of Dislocation |  |
| 131 | A qualitative study of quality of life domains and subdomains relevant to patients with spondyloarthritis |  |
| 132 | Problems faced at work due to inflammatory arthritis: new insights gained from understanding patients' perspective |  |
| 133 | Patient perspectives on health care provider practices leading to an axial spondyloarthritis diagnosis: an exploratory qualitative research study |  |
| 134 | Primary care physician perspectives on barriers to diagnosing axial Spondyloarthritis: a qualitative study |  |
| 135 | Primary care physician perspectives on screening for axial spondyloarthritis: A qualitative study |  |
| 136 | The Many Facets of Spine Pathology : a Review and Evaluation of Approaches in Biological Anthropology and Their Current and Future Applications |  |
| 137 | Experiences of group-based cognitive behavioural therapy for insomnia among patients with rheumatoid arthritis: a qualitative study |  |
| 138 | Usability and understandability of a web-based medical communication aid for patients with ankylosing spondylitis in South Korea A mixed-methods study |  |
| 139 | Predictors of functional deterioration in Chinese patients with Psoriatic arthritis: a longitudinal study |  |
| 140 | A Qualitative Study Exploring Participants' Perception of the Making It Work Program, an Online Program to Help People with Inflammatory Arthritis Maintain Employment |  |
| 141 | Impact of anti-tumour necrosis factor alpha treatment on admissions to hospital and days of sick leave in patients with ankylosing spondylitis |  |
| 142 | Learning Needs Assessment for Patients with Cancer and a Pre-Existing Autoimmune Disease Who Are Candidates to Receive Immune Checkpoint Inhibitors |  |
| 143 | Sacro-iliac joint scanning with technetium-99 diphosphonate |  |
| 144 | Quality control of radiographic imaging of the lumbar spine |  |
| 145 | Alexander Technique Lessons, Acupuncture Sessions or usual care for patients with chronic neck pain (ATLAS): study protocol for a randomised controlled trial |  |
| 146 | Perceived challenges at work and need for professional support among people with inflammatory arthritis-a qualitative interview study |  |
| 147 | Nonradiographic axial spondyloarthritis: expanding the spectrum of an old disease A narrative review |  |
| 148 | Matching prevalence of peripheral arthritis and acute anterior uveitis in individuals with ankylosing spondylitis |  |
| 149 | The journey to diagnosis in AS/axial SpA: the impact of delay |  |
| 150 | Disease severity and psychological status in ankylosing spondylitis |  |
| 151 | Role of advanced MRI techniques for sacroiliitis assessment and quantification |  |
| 152 | A Narrative Review of the Classification and Use of Diagnostic Ultrasound for Conditions of the Achilles Tendon |  |
| 153 | We Got Your Back! Help Care Seeking and Caregiving in Mexican Indigenous Men With Ankylosing Spondylitis |  |
| 154 | A narrative literature review of the impact of anti-TNFα treatment on the occupational performance of people with rheumatoid arthritis or ankylosing spondylitis |  |
| 155 | Better but not best: a qualitative exploration of the experiences of occupational gain for people with inflammatory arthritis receiving anti-TNFα treatment |  |
| 156 | Experiences of a patient-initiated self-monitoring service in inflammatory arthritis: A qualitative exploration |  |
| 157 | Systematic review of rheumatic disease epidemiology in the indigenous populations of Canada, the United States, Australia, and New Zealand |  |
| 158 | Clinical significance of specific spinal mobilization for patients with ankylosing spondylitis evaluated by quantitative assessments and patient interviews |  |
| 159 | Patients' experiences of fatigue in axialspondyloarthritis |  |
| 160 | Perceptions and Outcomes of Pregnancy and Lactation in Patients with Rheumatic Diseases |  |
| 161 | Rheumatology science and practice in India |  |
| 162 | Enthesitis-related arthritis: current perspectives |  |
| 163 | Non-pharmacological interventions and corticosteroid injections for the management of the Achilles tendon in inflammatory arthritis: a systematic review |  |
| 164 | Tofacitinib for the treatment of active ankylosing spondylitis in adults |  |
| 165 | Fatigue in Egyptian patients with rheumatic diseases: a qualitative study |  |
| 166 | Young people's perspectives on patient-reported outcome measures in inflammatory arthritis: Results of a multicentre European qualitative study from a EULAR task force |  |
| 167 | When you read this, you really feel old! Perspectives of young people with inflammatory arthritis on patient reported outcome measures from a european qualitative study |  |
| 168 | Prevalence and complication of COVID-19 in patients with ankylosing spondylitis (AS) and its relationship with TNF-a inhibitors |  |
| 169 | The patient experience with fatigue and content validity of a measure to assess fatigue severity: qualitative research in patients with ankylosing spondylitis (AS) |  |
| 170 | Strategies for the assessment of competences during rheumatology training across Europe: results of a qualitative study |  |
| 171 | Results of a specific smoking cessation program for patients with arthritis in a rheumatology clinic |  |
| 172 | The ASAS-OMERACT core domain set for axial spondyloarthritis |  |
| 173 | Recent updates in enthesitis-related arthritis |  |
| 174 | WEB-Based Physiotherapy for People With Axial Spondyloarthritis |  |
| 175 | Impact of a Gluten-free Diet on Quality of Life in Patients With Axial Spondyloarthritis |  |
| 176 | The Role of BCG Vaccine in the Clinical Evolution of COVID-19 and in the Efficacy of Anti-SARS-CoV-2 Vaccines |  |
| 177 | Problem specification of psychosocial and physical sequelae in ankylosing spondylitis: Phase I of a competency-based model of coping |  |
| 178 | A survey on acceptance of covid-19 vaccination among patients with rheumatic diseases -a single center experience in malaysia |  |
| 179 | The (cost-)effectiveness of PREPARE (Pre-pain rehabilitation) treatment, a Motivational interviewing (MI)-based nurse-led intervention on motivation and adherence for, and participation after pain rehabilitation treatment in chronic non-specific musculoskeletal pain syndrome patients: a randomized controlled trial (RCT) |  |
| 180 | Economic approaches to the problem of acceptable clinical risks: the case of prescription drugs and chronic rheumatic disease |  |
| 181 | Economic approaches to the problem of acceptable clinical risks: the case of prescription drugs and chronic rheumatic disease (clinical risk, rheumatoid arthritis) |  |
| 182 | Willingness to accept risk in the treatment of rheumatic disease |  |
| 183 | Prevalence of diagnosis and direct treatment costs of back disorders in 644,773 children and youths in Germany |  |
| 184 | Physical Activity and Exercise: Perspectives of Adults With Ankylosing Spondylitis |  |
| 185 | Patient perspectives on the pathway to psoriatic arthritis diagnosis: results from a web-based survey of patients in the United States |  |
| 186 | Patients suffering from rheumatic disease describing own experiences from participating in Basic Body Awareness Group Therapy: A qualitative pilot study |  |
| 187 | The effect of reproductive events and alterations of sex hormone levels on the symptoms of fibromyalgia |  |
| 188 | Lessons learned from pilot implementation of physical activity recommendations in axspa exercise group therapy-less may be more |  |
| 189 | Mapping the patient journey of nonradiographic axial spondyloarthritis: Perspective of professionals and patients |  |
| 190 | The journey of the non-radiographic axial spondyloarthritis patient: the perspective of professionals and patients |  |
| 191 | Performance of F-sodium fluoride positron emission tomography with computed tomography to assess inflammatory and structural sacroiliitis on magnetic resonance imaging in axial spondyloarthritis |  |
| 192 | Hypogonadism in men with inflammatory joint diseases: Frequency and clinical characteristics |  |
| 193 | Glass half full: A diary and interview qualitative investigation of flourishing among adolescents living with chronic pain |  |
| 194 | Assessing a child or adolescent with low back pain is different to assessing an adult with low back pain |  |
| 195 | Web-based physiotherapy for people with axial spondyloarthritis (WEBPASS) - a study protocol |  |
| 196 | Online physiotherapy for people with axial spondyloarthritis: quantitative and qualitative data from a cohort study |  |
| 197 | Qualitative interview study exploring the patient experience of living with axial spondyloarthritis and fatigue: difficult, demanding and draining |  |
| 198 | Development of the Warwick Axial Spondyloarthritis faTigue and Energy questionnaire (WASTEd)-a new patient-reported outcome measure |  |
| 199 | Feasibility, acceptability and change in health following a telephone-based cognitive behaviour therapy intervention for patients with axial spondyloarthritis |  |
| 200 | Not-belonging': illness narratives of Mexican patients with ankylosing spondylitis |  |
| 201 | Sexual function and reproduction can be impaired in men with rheumatic diseases: A systematic review |  |
| 202 | Quantitative measurement of patient status in the regular care of patients with rheumatic diseases over 25 years as a continuous quality improvement activity, rather than traditional research |  |
| 203 | Exercise behaviour in ankylosing spondylitis |  |
| 204 | Patients' Views on Routine Collection of Patient-Reported Outcomes in Rheumatology Outpatient Care: A Multicenter Focus Group Study |  |
| 205 | Blood-based biomarkers of chronic inflammation |  |
| 206 | Qualitative assessment of medication adherence in patients with rheumatic diseases on biologic therapy |  |
| 207 | Exploring strategies to support medication adherence in patients with inflammatory arthritis: a patient-oriented qualitative study using an interactive focus group activity |  |
| 208 | Diagnostic tools and strategies for assessing disease progression in Alkaptonuria |  |
| 209 | Impact of a motivational intervention on coping with chronic pain.: Results of a controlled efficacy study |  |
| 210 | Identification of similarities and differences in functioning in persons with rheumatoid arthritis and ankylosing spondylitis using the International Classification of Functioning, Disability and Health (ICF) |  |
| 211 | Understanding beliefs related to physical activity in people living with axial Spondyloarthritis: a theory-informed qualitative study |  |
| 212 | (Un)Spoken realities of living with axial spondyloarthritis: a qualitative study focused on couple experiences |  |
| 213 | A concept elicitation study to understand the relationship between sleep and pain in rheumatoid arthritis and axial spondyloarthritis |  |
| 214 | I am still bed six: a collection of poetry, and, poetry as therapy and poetry beyond therapy |  |
| 215 | Influence of clinical information on the diagnostic validity of MRI in the detection of abacterial sacroiliitis |  |
| 216 | Patients' experiences regarding self-monitoring of the disease course: An observational pilot study in patients with inflammatory rheumatic diseases at a rheumatology outpatient clinic in the Netherlands |  |
| 217 | Systematic Review of the Impact of Inflammatory Arthritis on Intimate Relationships and Sexual Function |  |
| 218 | Occupational therapy assessments and interventions for patients with ankylosing spondylitis: a scoping review protocol |  |
| 219 | Prevalence and clinical characteristics of psoriasis in spondyloarthritis: a descriptive analysis of 275 patients |  |
| 220 | Living with ankylosing spondylitis: an open response survey exploring physical activity experiences |  |
| 221 | Rheumatology care of migrants from sub-Saharan Africa: a literature review and qualitative pilot study of patients' perspectives |  |
| 222 | Rheumatic manifestations of psoriasis |  |
| 223 | Exploring associations with depressive and anxiety symptoms among Syrian patients with ankylosing spondylitis undergoing biological treatment: A cross-sectional study |  |
| 224 | TNF-alpha Blockers Showed Prophylactic Effects in Preventing COVID-19 in Patients with Rheumatoid Arthritis and Seronegative Spondyloarthropathies: A Case-Control Study |  |
| 225 | Efficacy and safety of the optimisation of biological therapy in non-infectious uveitis: Systematic review |  |
| 226 | Musculoskeletal manifestations in a population-based cohort of inflammatory bowel disease patients |  |
| 227 | Health-related quality of life among spondyloarthritis and chronic low back pain patients: results from a nationwide population-based survey |  |
| 228 | Patient perceptions, attitudes and concerns about anti-tnf drugs |  |
| 229 | Evaluation of the novel ultrasound score for large joints in psoriatic arthritis and ankylosing spondylitis: six month experience in daily clinical practice |  |
| 230 | Cervical spine alignment, sagittal deformity, and clinical implications A review |  |
| 231 | Cardiovascular disease in spondyloarthritis: a narrative review of risk factors and the effect of treatments |  |
| 232 | Analysis of phenotypical correlations between qualitative traits. II. Evaluation of the contribution of the nominal factor to variations in susceptibility to multifactorial diseases |  |
| 233 | Rheumatic disease patient decision-making about COVID-19 vaccination: a qualitative analysis |  |
| 234 | Development of a screening tool to identify patients with axial spondyloarthritis: a cognitive interview study |  |
| 235 | Let's talk about inflammatory back pain: a qualitative study regarding experiences of disclosure in people with ankylosing spondylitis/axial spondyloarthritis |  |
| 236 | Biological and synthetic target DMARDs in psoriatic arthritis |  |
| 237 | Thirty-six years experience of cervical extension osteotomy in ankylosing spondylitis: techniques and outcomes |  |
| 238 | Investigating Dimensions of Stiffness in Rheumatoid and Psoriatic Arthritis: The Australian Rheumatology Association Database Registry and OMERACT Collaboration |  |
| 239 | Concepts important to patients with psoriatic arthritis are not adequately covered by standard measures of functioning |  |
| 240 | It's magic stuff': the experiences of patients with ankylosing spondylitis taking anti-TNF-alpha medication |  |
| 241 | An Exploration of the Impact of Anti-TNFα Medication on Exercise Behaviour in Patients with Ankylosing Spondylitis |  |
| 242 | Patient Burden of Axial Spondyloarthritis |  |
| 243 | Exploring Family Planning, Parenting, and Sexual and Reproductive Health Care Experiences of Men With Rheumatic Diseases |  |
| 244 | Prevalence and Characteristics of Flare-ups of Chronic Nonspecific Back Pain in Primary Care A Telephone Survey |  |
| 245 | Immune-mediated lung diseases: A narrative review |  |
| 246 | Temporomandibular joint disorders in seronegative spondyloarthritis: what a rheumatologist needs to know? |  |
| 247 | Improved precision of syndesmophyte measurement for the evaluation of ankylosing spondylitis using CT: a phantom and patient study |  |
| 248 | Gender perspective in clinical epidemiology. Learning from spondyloarthritis |  |
| 249 | Education and learning for people with ankylosing spondylitis |  |
| 250 | Living well with chronic pain: a classical grounded theory |  |
| 251 | Reactive arthritis and other musculoskeletal symptoms associated with acquisition of diarrhoeagenic Escherichia coli (DEC) |  |
| 252 | Patients perspectives on self-management of axial spondyloarthritis fatigue |  |
| 253 | Problems of rheumatoid arthritis and ankylosing spondylitis patients in their labor and life environments |  |
| 254 | Certolizumab Pegol Treatment in Patients with Axial-Spondyloarthritis-Associated Acute Anterior Uveitis: a Narrative Review |  |
| 255 | Patients’ Needs Concerning Patient Education in Axial Spondyloarthritis: A Qualitative Study |  |
| 256 | Cardiometabolic risk factors as determinants of peripheral nerve function: the Maastricht Study |  |
| 257 | Identification of the most common problems by patients with ankylosing spondylitis using the international classification of functioning, disability and health |  |
| 258 | Clinical utility of therapeutic drug monitoring in biological disease modifying anti-rheumatic drug treatment of rheumatic disorders: a systematic narrative review |  |
| 259 | Are globals for health, well-being and quality of life interchangeable? A mixed methods study in ankylosing spondylitis patients and controls |  |
| 260 | Biosimilar, so it looks alike, but what does it mean? A qualitative study of Danish patients' perceptions of biosimilars |  |
| 261 | Motivations and objections to implement a spondyloarthritis integrated care pathway. A qualitative study with primary care physicians |  |
| 262 | A qualitative study of the impact of Crohn's disease from a patient's perspective |  |
| 263 | A review of 100 patients with ankylosing spondylitis with particular reference to socio-economic effects |  |
| 264 | Prevalence of seronegative spondyloarthritis in the army force of China |  |
| 265 | Symptom Appraisal and Help-Seeking Among Patients With Autoimmune Rheumatic Diseases: A Qualitative Study |  |
| 266 | Social cognitive theory to improve symptom appraisal and help-seeking among patients with autoimmune rheumatic diseases: A qualitative study |  |
| 267 | Monoclonal antibodies for chronic pain: a practical review of mechanisms and clinical applications |  |
| 268 | Quality of life in ankylosing spondylitis |  |
| 269 | Person-centered Art Therapy Impact on Depression and Self-Esteem: A Case Study in Patients with Ankylosing Spondylitis |  |
| 270 | Systematic Literature Review of the Use of Productivity Losses/Gains in Cost-Effectiveness Analyses of Immune-Mediated Disorders |  |
| 271 | Psoriatic Spondylitis: A Disease Manifestation in Debate Evidences to Know for the Clinical Rheumatologist |  |
| 272 | Biosimilars: From Extrapolation into Off Label Use |  |
| 273 | The mSQUASH; a valid, reliable and responsive questionnaire for daily physical activity in patients with axial spondyloarthritis |  |
| 274 | Prevalence survey of rheumatoid arthritis and spondyloarthropathy in Lithuania |  |
| 275 | Effect of TNF-inhibitor therapy on spinal structural progression in ankylosing spondylitis patients: A systematic review and meta-analysis |  |
| 276 | Ankylosing spondylitis in Northern Jordan |  |
| 277 | A comparison of telephone and paper self-completed questionnaires of main patient-related outcome measures in patients with ankylosing spondylitis and psoriatic arthritis |  |
| 278 | Anti-IL-17 Agents in the Treatment of Axial Spondyloarthritis |  |
| 279 | Meloxicam in the management of post-operative pain: Narrative review |  |
| 280 | Prevalence of spondyloarthropathies in Alaskan Eskimos |  |
| 281 | Secukinumab in axial spondyloarthritis: a narrative review of clinical evidence |  |
| 282 | Ankylosing spondylitis and heart abnormalities: do cardiac conduction disorders, valve regurgitation and diastolic dysfunction occur more often in male patients with diagnosed ankylosing spondylitis for over 15 years than in the normal population? |  |
| 283 | Prevalence, demographics, and clinical characteristics of Latin American patients with spondyloarthritis |  |
| 284 | Balneotherapy in chronic inflammatory rheumatic diseases-a narrative review |  |
| 285 | What can the periodontal community learn from the pathophysiology of rheumatoid arthritis? |  |
| 286 | Mental health status and leisure-time physical activity contribute to fatigue intensity in patients with spondylarthropathy |  |
| 287 | Therapeutic exercises and rehabilitation in axial spondyloarthropathy: Balancing benefits with unique challenges in the Asia-Pacific countries |  |
| 288 | Human Leukocyte Antigen B27-Negative Axial Spondyloarthritis: What Do We Know? |  |
| 289 | Articular manifestations in inflammatory bowel disease patients: a prospective study |  |
| 290 | Diagnosis delay in patients with ankylosing spondylitis: possible reasons and proposals for new diagnostic criteria |  |
| 291 | The Juvenile Arthritis Quality of Life Questionnaire--development of a new responsive index for juvenile rheumatoid arthritis and juvenile spondyloarthritides |  |
| 292 | Management of spinal fractures in patients with ankylosing spondylitis |  |
| 293 | Spondyloarthropathies in circumpolar populations of Chukotka (Eskimos and Chukchi): epidemiology and clinical characteristics |  |
| 294 | A Review of the Safety of Interleukin-17A Inhibitor Secukinumab |  |
| 295 | High disease activity is related to low levels of physical activity in patients with ankylosing spondylitis |  |
| 296 | The role for JAK inhibitors in the treatment of immune-mediated rheumatic and related conditions |  |
| 297 | Advantages of remote monitoring the activity of patients with axial spondylitis (progress study) |  |
| 298 | Sexual activity in ankylosing spondylitis |  |
| 299 | Diffuse Idiopathic Skeletal Hyperostosis and Ankylosing Spondylitis: A Challenging Case and Review of the Literature |  |
| 300 | Perioperative medical management for patients with RA, SPA, and SLE undergoing total hip and total knee replacement: a narrative review |  |
| 301 | Rehabilitation of patients with spondyloarthritis: a narrative review |  |
| 302 | Analysis of the Literature on Cervical Spine Fractures in Ankylosing Spinal Disorders |  |
| 303 | Response to one infusion predicts subsequent improvement as well as the rate of relapse of ankylosing spondylitis infused with three pulses of infliximab |  |
| 304 | Benefits of tumor necrosis factor inhibitors for cardiovascular disease in ankylosing spondylitis |  |
| 305 | Exploring the Future Role of Self-Tracking Data in the Rheumatology Clinic |  |
| 306 | Using qualitative methods for a conceptual analysis of measures of health status and presenteeism prior to a mapping study |  |
| 307 | Reliability of the Canadian Occupational Performance Measure in patients with ankylosing spondylitis |  |
| 308 | Ankylosing spondylitis in Singaporean Chinese--a clinical profile |  |
| 309 | Impact of sex and gender on axSpA diagnosis and outcomes |  |
| 310 | Interleukin-32 as a biomarker in rheumatic diseases: A narrative review |  |
| 311 | Different drug survival of first line tumour necrosis factor inhibitors in radiographic and non-radiographic axial spondyloarthritis: a multicentre retrospective survey |  |
| 312 | Nonradiographic axial spondyloarthritis: expanding the spectrum of an old disease: A narrative review |  |
| 313 | Recognizing Axial Spondyloarthritis: A Guide for Primary Care |  |
| 314 | Human resting muscle tone (HRMT): narrative introduction and modern concepts |  |
| 315 | Pregnancy and early onset pauciarticular juvenile chronic arthritis |  |
| 316 | Etiology and treatment of cervical kyphosis: state of the art review-a narrative review |  |
| 317 | Prevalence of ankylosing spondylitis and related spondyloarthritides in an urban area of Izmir, Turkey |  |
| 318 | Quality of Care in Rheumatoid Disease from the Clinician Perspective: A Modified Delphi Panel Approach |  |
| 319 | Patterns of dyslipidemia in young patients with seronegative spondyloarthropathies without cardiovascular diseases |  |
| 320 | Ocular Manifestations of Spondyloarthritis |  |
| 321 | Autoimmune rheumatic diseases associated with granulomatous mastitis |  |
| 322 | FDA-Approved Janus Kinase-Signal Transducer and Activator of Transcription (JAK-STAT) Inhibitors for Managing Rheumatoid Arthritis: A Narrative Review of the Literature |  |
| 323 | Novel insights into the anatomy and histopathology of the sacroiliac joint and correlations with imaging signs of sacroiliitis in case of axial spondyloarthritis |  |
| 324 | Treatment with biological therapy is associated with faster recovery and lower frequency of treatment switch among rheumatic patients with Chikungunya fever |  |
| 325 | Psoriatic arthritis and ankylosing spondylitis impact on health-related quality of life and working life: a comparative population-based study |  |
| 326 | Insight into the Quality of Life of Patients with Ankylosing Spondylitis: Real-World Data from a US-Based Life Impact Survey |  |
| 327 | Prevalence of spondyloarthropathies in France: 2001 |  |
| 328 | A Road Map of the Axial Spondyloarthritis Continuum |  |
| 329 | Male Fertility in Spondyloarthritis: from Clinical Issues to Cytokines Milieu. A Narrative Review |  |
| 330 | Targeting cytokines in reduction of depressive symptoms: A comprehensive review |  |
| 331 | Brazilian-Portuguese version and applicability questionnaire of the mobility index for ankylosing spondyliti |  |
| 332 | Relationship of environmental exposures and ankylosing spondylitis and spinal mobility: US NHAENS, 2009-2010 |  |
| 333 | Matrix Metalloproteinases; A Biomarker of Disease Activity and Prognosis in Spondyloarthritis: A Narrative Review |  |
| 334 | Vertebral osteomyelitis: long-term disability assessment and prognostic factors |  |
| 335 | Performance of different criteria sets for inflammatory back pain in patients with axial spondyloarthritis with and without radiographic sacroiliitis |  |
| 336 | Telephone interview strategy can be used for screening inflammatory back pain in the community |  |
| 337 | Impact of Secukinumab on Patient-Reported Outcomes in the Treatment of Ankylosing Spondylitis: Current Perspectives |  |
| 338 | Calcium physiology, metabolism and supplementation: a glance at patients with ankylosing spondylitis |  |
| 339 | Spondyloarthropathies That Mimic Ankylosing Spondylitis: A Narrative Review |  |
| 340 | Seronegative spondyloarthropathies in Greece: a population-based study of prevalence, clinical pattern, and management. The ESORDIG study |  |
| 341 | No appreciable decrease in fertility in Behçet's syndrome |  |
| 342 | Effect of biological therapy on work participation in patients with ankylosing spondylitis: a systematic review |  |
| 343 | Adverse events in patients with ankylosing spondylitis treated with TNF inhibitors: a cross-sectional study |  |
| 344 | Psoriatic Spondylitis: A Disease Manifestation in Debate: Evidences to Know for the Clinical Rheumatologist |  |
| 345 | Treatment adherence and disease burden of individuals with rheumatic diseases admitted as outpatients to a large rheumatology center in Shanghai, China |  |
| 346 | Defining and managing flares in axial spondyloarthritis |  |
| 347 | Therapeutic Potential of IL-1 Antagonism in Hidradenitis Suppurativa |  |
| 348 | Assessment of relationship between beliefs' about medicines and treatment adherence in ankylosing spondylitis patients |  |
| 349 | Communication strategies are highly important to avoid nocebo effect when performing non-medical switch from originator product to biosimilar product: Danish results from applying the Parker model a qualitative 3-step research model |  |
| 350 | NICE guidance on Spondyloarthritis for over 16s: management and research recommendations relevant to physiotherapists |  |
| 351 | Patient perceptions on switching from reference product adalimumab to biosimilar adalimumab-atto |  |
| 352 | Brave Men and "Emotional Women": A Theory-Guided Literature Review on Gender Bias in Health Care and Gendered Norms towards Patients with Chronic Pain |  |
| 353 | An exploration of patients' experiences of fatigue in axial spondyloarthitis |  |
| 354 | 甲氨蝶呤治疗类风湿关节炎的跌宕历程 |  |
| 355 | 人工全髋关节翻修原因及方法临床分析 |  |
| 356 | 理性情绪疗法对强直性脊柱炎患者情绪与疼痛的干预研究 |  |
| 357 | PKP术后早期对OVCF患者脊柱--骨盆矢状面平衡状态影响的研究 |  |
| 358 | 醋甲唑胺治疗强直性脊柱炎 |  |
| 359 | 周乃玉学术思想和临床经验总结及健脾益气治疗干燥综合征临床研究 |  |
| 360 | 低剂量斜冠状位骶髂关节CT检查在强直性脊柱炎患者诊断中的潜在临床价值分析 |  |
| 361 | 薄氏腹针配合药物疗法治疗强直性脊柱炎的临床疗效评价 |  |
| 362 | 中药热敷合手法弹拔压腿锻炼治疗膝骨关节炎的临床研究 |  |
| 363 | 神经病理性疼痛评估工具用于痛性糖尿病周围神经病变患者的评价 |  |
| 364 | 复合骨髓基质干细胞的β-磷酸三钙在兔脊柱后外侧融合中的应用研究 |  |
| 365 | 中医诊治痹证学术源流探讨及文献整理与资料查询系统建立 |  |
| 366 | 基于半结构式访谈与扎根理论强直性脊柱炎中医辨治名家经验 |  |
| 367 | 独活寄生合剂对激素性股骨头缺血坏死防治作用的研究 |  |
| 368 | 氯化矢车菊素治疗溶骨性疾病的机制研究 |  |
| 369 | 胸腰段后凸畸形影响腰椎诸节段矢状面稳定性的研究进展 |  |
| 370 | 雷公藤治疗强直性脊柱炎的研究现状 |  |
| 371 | 中药通痹灵片治疗强直性脊柱炎患者的生存质量研究 |  |
| 372 | 中轴型脊柱关节炎患者疼痛自我管理现状及影响因素 |  |
| 373 | 健脾清热通络方调控NONHSAT227927.1/JAK2/STAT3组合改善强直性脊柱炎湿热痹阻证患者感受的机制研究 |  |
| 374 | 颈椎强直性脊柱炎骨折脱位临床病例分析 |  |
| 375 | 强直性脊柱炎（大（尢娄））证候研究及与血清黏附分子的相关性初步探讨 |  |
| 376 | 基于文献计量的中医治疗强直性脊柱炎的知识图谱研究 |  |
| 377 | 双节段、单节段去松质骨截骨与全脊柱截骨强直性脊柱后凸矫形策略的有限元分析 |  |
| 378 | 髋臼横韧带与髋臼卵圆窝顶点作为解剖标志对髋臼假体植入的参照作用 |  |
| 379 | 甲芬那酸通过抑制慢性应激诱导的小胶质细胞激活来缓解神经炎症并改善抑郁症状 |  |
| 380 | 基于单细胞测序探究强直性脊柱炎炎症和骨化进程中巨噬细胞和干细胞的交互关系 |  |
| 381 | 强直性脊柱炎骶髂关节CT分级标准比较与低剂量斜冠状位扫描技术的应用价值研究 |  |
| 382 | 低剂量斜冠状位骶髂关节CT检查诊断强直性脊柱炎的研究初探 |  |
| 383 | 低剂量斜冠状位骶髂关节CT在强直性脊柱炎诊断中的应用价值研究 |  |
| 384 | 通痹灵片治疗强直性脊柱炎近期疗效观察 |  |
| 385 | CNTFR在强直性脊柱炎中的表达变化及其对成骨细胞活性的影响 |  |
| 386 | 保留股骨颈型髋关节假体周围骨折原因的回顾性分析 |  |
| 387 | 强直性脊柱炎的中医治疗研究进展与展望 |  |
| 388 | 强直性脊柱炎合并葡萄膜炎的临床研究 |  |
| 389 | 股髋撞击综合征的影像学表现及临床应用研究 |  |
| 390 | 抗TNF-α小分子抑制剂的研究及抗CD20Fab-TNFα融合蛋白表达载体的构建、表达和活性的初步测定 |  |
| 391 | 名医林应强教授诊治早期强直性脊柱炎经验撷英 |  |
| 392 | IL-1基因多态性与中国AS关联分析及其在AS患者的表达研究 |  |
| 393 | 针灸治疗强直性脊柱炎临床进展 |  |
| 394 | 补肾壮督、活血通络法治疗强直性脊柱炎肾虚血瘀证临床研究 |  |
| 395 | 护理干预对强直性脊柱炎患者服药依从性影响的研究 |  |
| 396 | 国医大师韦贵康“3+X”疗法治疗早期强直性脊柱炎经验浅析 |  |
| 397 | 补阳还五汤治疗中医骨伤科疾病研究进展 |  |
| 398 | 近五年国内经筋学术论文文献计量学研究 |  |
| 399 | 强直性脊柱炎患者主观幸福感现状及影响因素分析 |  |
| 400 | 强直性脊柱炎与心理社会因素相关性调查 |  |
| 401 | 基于静息态功能磁共振和VBM的强直性脊柱炎伴腰背部疼痛患者脑功能及结构的研究 |  |
| 402 | 强直性脊柱炎胸腰椎后凸畸形截骨矫形术围手术期并发症及危险因素分析 |  |
| 403 | 强直性脊柱炎患者生活质量与临床及影像学参数的相关性 |  |
| 404 | 强直性脊柱炎与HLA-B27基因亚型动态定量表达的相关性研究 |  |
| 405 | 李彦民治疗强直性脊柱炎经验浅谈 |  |
| 406 | 中轴型脊柱关节炎患者疼痛特征及其与疾病活动度和功能状况的关系 |  |
| 407 | 浅谈强直性脊柱炎相关生物制剂治疗进展 |  |
| 408 | 中医分期治疗强直性脊柱炎的临床研究 |  |
| 409 | 郭立中教授从扶阳论治风寒湿痹的临床经验研究 |  |
| 410 | 强直性脊柱炎患者希望水平现状调查 |  |
| 411 | 阎小萍教授风湿病学术思想和基于“寒热为纲”治疗强直性脊柱炎的临床研究 |  |
| 412 | 严重骨质疏松症患者全髋关节置换术后临床疗效观察 |  |
| 413 | 何羿婷教授治疗强直性脊柱炎的用药规律研究 |  |
| 414 | 从毒瘀论治强直性脊柱炎的临床研究 |  |
| 415 | 应用多维数据分析规范强直性脊柱炎的中医辨证规律 |  |
| 416 | 中医体质与强直性脊柱炎患者缓解期功能的相关性研究 |  |
| 417 | 强直性脊柱炎早中期腰背痛的中医干预研究 |  |
| 418 | 强直性脊柱炎患者生物制剂注射过程中的问题探讨 |  |
| 419 | 强直性脊柱炎早期骶髂关节炎的高场MRI评价 |  |
| 420 | 与汉族强直性脊柱炎相关的非MHC区域基因的单核苷酸多态性在急性前葡萄膜炎中的遗传易感性研究 |  |
| 421 | 基于古今医案数据分析的强直性脊柱炎的证治规律研究 |  |
| 422 | 流式细胞技术检测人HLA-B27抗原诊断强直性脊柱炎研究 |  |
| 423 | 李堪印教授“从筋论治”强直性脊柱炎学术思想整理研究 |  |
| 424 | 强直性脊柱炎患者出院准备度现状及影响因素分析 |  |
| 425 | 补肾疏肝养血法对强直性脊柱炎伴抑郁状态的疗效观察 |  |
| 426 | 基于文献数据挖掘的中医药治疗强直性脊柱炎血瘀证用药规律 |  |
| 427 | 基于网络药理学和代谢组学技术的南蛇藤提取物抗类风湿关节炎作用机制研究 |  |
| 428 | 中医药治疗强直性脊柱炎的系统评价及用药分析 |  |
| 429 | 中轴型脊柱关节炎患者抗风湿药物服药信念现状及其影响因素 |  |
| 430 | 通痹灵治疗强直性脊柱炎的临床研究近斯疗效评价 |  |
| 431 | 全髋关节置换术后利伐沙班预防深静脉血栓形成的研究 |  |
| 432 | 中医药辨证论治辅助治疗AS的临床研究 |  |
| 433 | 传统国学教育对强直性脊柱炎患者自我感受及生活质量的影响 |  |
| 434 | 强直性脊柱炎的护理体会 |  |
| 435 | 补肾活血法治疗强直性脊柱炎临床研究及抗骨化作用实验探讨 |  |
| 436 | 基于半结构式访谈与扎根理论强直性脊柱炎中医辨治名家经验 |  |
| 437 | 强直性脊柱炎的中医证治探讨 |  |
| 438 | 不明原因腰背痛不竞忽视强直性脊柱炎——湖南中医药大学第一附属医院王莘智访谈 |  |
| 439 | 计算机导航辅助颈椎椎弓根螺钉内固定技术 |  |
| 440 | 中药临床药学服务内容探索及中药治疗强直性脊柱炎的临床用药与Meta分析 |  |
| 441 | 陈湘君教授风湿病学术思想总结及阴虚内热证系统性红斑狼疮相关影响因素研究 |  |
| 442 | Th17细胞在银屑病发病中的作用及意义研究 |  |
| 443 | 清热滋阴活血方治疗自体免疫性前葡萄膜炎临床与实验研究 |  |
| 444 | 颈椎过伸性损伤的特点及分型应用的相关研究 |  |
| 445 | 强直性脊柱炎2218例临床资料分析及中医证候研究 |  |
| 446 | 冯兴华教授痹证学术思想及从肝论治痹证法治疗偏头痛的临床研究 |  |
| 447 | 全髋关节置换术治疗非功能位髋关节骨性强直12例17髋 |  |
| 448 | 椎体钉重建脊柱前方稳定性的失误与预防 |  |
| 449 | 国医大师朱良春应用奇经八脉理论治疗强直性脊柱炎学术思想研究 |  |
| 450 | 积极治疗,“强直”不过是“纸老虎” |  |
| 451 | 强直性脊柱炎患者治疗依从性的研究进展 |  |
| 452 | SOST和β-catenin在强直性脊柱炎外周关节滑膜组织中骨化作用机制研究 |  |
| 453 | 强直性脊柱炎患者心理状态调查与分析 |  |
| 454 | 腰脊神经后支松解术治疗椎管外源性腰痛的临床观察与机制探讨 |  |
| 455 | 强直性脊柱炎患者在线健康信息寻求行为的质性研究 |  |
| 456 | HLA-B27检测对强直性脊柱炎诊断的价值 |  |
| 457 | ITP中自身免疫性初始B细胞的积累及CARD9 SNP rs4077515与ITP易感性的关系研究 |  |
| 458 | 强直性脊柱炎胸腰段后凸畸形矫形设计 |  |
| 459 | 乳腺癌术后辅助化疗不良反应以及中医药治疗综述 |  |
| 460 | 基于数据挖掘探讨周翠英教授治疗强直性脊柱炎的经验 |  |
| 461 | 基于足太阳经筋病灶点关联分析的针刺治疗强直性脊柱炎临床研究 |  |
| 462 | 基于文献的艾灸疗法应用规律研究 |  |
| 463 | 生长因子Progranulin结合肿瘤坏死因子受体家族成员及其在自身免疫性疾病中的功能研究 |  |
| 464 | 强直性脊柱炎患者骨质疏松症与中医辨证分型的关系研究 |  |
| 465 | 马来西亚及新加坡名中医治疗痹症特色疗法及经验整理 |  |
| 466 | 中药（强脊1号）对活动期强直性脊柱炎作用的临床研究 |  |
| 467 | 股骨头坏死误诊相关因素及中医证候分析 |  |
| 468 | 吴中朝教授火针治痹经验总结及膝骨关节炎火针扬刺临床研究 |  |
| 469 | 李艳传承国医大师李济仁论治骨痹之思路与方法 |  |
| 470 | 强直性脊柱炎的中医证治规律研究 |  |
| 471 | 综合护理干预对强直性脊柱炎患者的功能锻炼依从性及睡眠质量的影响研究 |  |
| 472 | 强直性脊柱炎髋关节病变小针刀规范化操作探讨 |  |
| 473 | 阎小萍教授风湿病学术思想及治疗强直性脊柱炎学术经验与临床研究 |  |
| 474 | 补肾强督法对AS患者骨质疏松、骨量减少影响的临床研究 |  |
| 475 | 补肾强督方治疗强直性脊柱炎患者的临床研究及机制探讨 |  |
| 476 | 基于古今医案云平台系统挖掘邱磷安教授辨治强直性脊柱炎的临床经验 |  |
| 477 | 强直性脊柱炎患者生存质量现状及影响因素分析 |  |
| 478 | 强直性脊柱炎患者生存质量及影响因素分析 |  |
| 479 | 民族医药治疗强直性脊柱炎研究进展 |  |
| 480 | 内质网应激相关基因在自身免疫性疾病中的表达 |  |
| 481 | 基于古今医案数据分析的历节病证治规律研究 |  |
| 482 | 强直性脊柱炎髋关节CT征象研究 |  |
| 483 | 枸杞子临床应用及其用量 |  |
| 484 | 僵硬膝或强直膝关节行人工全膝关节置换的效果及关节活动范围影响分析 |  |
| 485 | 强直性脊柱炎患者股骨密度与腰椎椎体骨折的相关性 |  |
| 486 | 中西医结合治疗1275例全膝人工关节置换手术经验总结 |  |
| 487 | 医结合治疗1275例全膝人工关节置换手术经验总结 |  |
| 488 | 强直性脊柱炎患者疾病不确定感及影响因素分析 |  |
| 489 | 自身免疫抗体噬菌体库的构建及初步鉴定 |  |
| 490 | Th17/Treg在骨科疾病中的研究进展 |  |
| 491 | 王上增教授运用温肾通督法治疗强直性脊柱炎的经验总结 |  |
| 492 | 银质针治疗强直性脊柱炎的临床疗效研究 |  |
| 493 | 青海不同民族MICA-TM基因多态性与胃癌相关性分析 |  |
| 494 | 干眼的药物治疗 |  |
| 495 | 陶瓷—陶瓷假体摩擦界面的人工全髋关节中期临床疗效随访研究 |  |
| 496 | 氨甲环酸减少同期双侧全髋关节置换围手术期失血量的临床疗效分析 |  |
| 497 | 87例强直性脊柱炎患者生存质量现状 |  |
| 498 | 复方抗风湿药酒制备工艺、质量标准及稳定性研究 |  |
| 499 | 通导督脉针刺治疗强直性脊柱炎随机平行对照研究 |  |
| 500 | 父母育龄及胎次效应对强直性脊柱炎发病的影响 |  |
| 501 | 平衡火罐配合火龙灸在强直性脊柱炎中的应用研究 |  |
| 502 | 鹿角胶临床应用及其用量探究 |  |
| 503 | 阎小萍教授辨治强直性脊柱炎（大（尢娄））经验探析-附320例病例分析 |  |
| 504 | 思维导图在强直性脊柱炎患者健康管理中的应用研究 |  |
| 505 | 强直性脊柱炎患者对运动疗法的认知及护理干预的临床研究 |  |
| 506 | 基于文献计量学探讨蜂针疗法疾病谱研究 |  |
| 507 | 全身糖皮质激素治疗强直性脊柱炎证据不足? |  |
| 508 | 基于赋能理论的健康教育对强直性脊柱炎患者自我效能及生活质量的影响 |  |
| 509 | 关节病型银屑病临床与实验研究 |  |
| 510 | 髁限制性假体在复杂膝关节初次置换中近期临床疗效 |  |
| 511 | 耕耘不息 奋斗不已 |  |
| 512 | 基于文献整理和数据挖掘的解痉舒督汤治疗强直性脊柱炎临床研究 |  |
| 513 | 郭永昌治疗强直性脊柱炎经验介绍 |  |
| 514 | 强直性脊柱炎的中西医治疗进展 |  |
| 515 | 益肾温督法治疗强直性脊柱炎的临床疗效观察 |  |
| 516 | 司库奇尤单抗与阿达木单抗治疗强直性脊柱炎的疗效和安全性对比分析 |  |
| 517 | 补肾强督治偻汤对强直性脊柱炎患者骨质变化机理的研究 |  |
| 518 | 现代名老中医治疗强直性脊柱炎的经验 |  |
| 519 | 继承与创新中医药特色防治强直性脊柱炎 |  |
| 520 | 通痹汤治疗强直性脊柱炎的临床疗效观察 |  |
| 521 | 张鸣鹤教授麻醉下关节牵拉矫形经验介绍 |  |
| 522 | 周乃玉学术思想和临床经验总结及“痹玉康Ⅰ号”方治疗中晚期寒湿痹阻型类风湿关节炎的临床研究 |  |
| 523 | 补肾强督方对强直性脊柱炎（大（尢娄））患者骨破坏相关因子影响的研究 |  |
| 524 | 补肾强督方治疗强直性脊柱炎肾虚督寒血瘀证临床研究 |  |
| 525 | 血小板衍生生长因子-BB与腰椎黄韧带增生肥厚的相关性研究 |  |
| 526 | 张鸣鹤教授“清热活血”法论治强直性脊柱炎经验 |  |
| 527 | 乳腺癌患者人格、应对方式与活动参与功能和生命质量关系的研究 |  |
| 528 | 当代医家论治强直性脊柱炎经验探析 |  |
| 529 | 强直性脊柱炎患者功能锻炼体验的质性研究 |  |
| 530 | 强直性脊柱炎伴严重脊柱后凸畸形患者术中体位的护理 |  |
| 531 | DWI及DCE-MRI对强直性脊柱炎骶髂关节活动性病变评价的临床应用 |  |
| 532 | 强直性脊柱炎验案3则 |  |
| 533 | 强直性脊柱炎中医证候的研究 |  |
| 534 | 石家庄市骨痹(骨关节炎)证候类型调查分析 |  |
| 535 | 刘时觉教授治痹理论及其在强直性脊柱炎诊治中的运用 |  |
| 536 | 补肾强督法治疗强直性脊柱炎的疗效及抗骨化作用初探 |  |
| 537 | 基于综合转变模型的成人初显期强直性脊柱炎患者自我管理模式的构建 |  |
| 538 | 成人初显期强直性脊柱炎患者自我管理障碍因素的质性研究 |  |
| 539 | 强直性脊柱炎合并骨质疏松的中医证治规律研究 |  |
| 540 | 广西壮族强直性脊柱炎HLA-B27基因亚型分布研究 |  |
| 541 | 摆脱绝症，协调异常：强直性脊柱炎患者心理适应和生活意义重构 |  |
| Reason for exclusion: Reports not retrieved. (n=2) | |  |
| 1 | [An interview with Alan Ebringer. What is going to happen tomorrow concerning HLA-B27 and ankylosing spondylathritis? Interview by P. Youinou] |  |
| 2 | Doctor-patient communication in patients with arthritis |  |
| Reason for exclusion: The target population does not match. (n=6) | |  |
| 1 | Content validity of the ASQoL for use in a non-radiographic axial spondyloarthritis population: a qualitative study. | |
| 2 | Perceptions, attitudes and experiences of family caregivers of patients with musculoskeletal diseases: a qualitative approach. | |
| 3 | Dose reduction of biologic therapy in inflammatory arthritis: A qualitative study of patients' perceptions and needs. | |
| 4 | General Practitioners' Perceptions of Their Ability to Identify and Refer Patients with Suspected Axial Spondyloarthritis: A Qualitative Study. | |
| 5 | If three of my brothers have ankylosing spondylitis, why does the doctor say it is not necessarily hereditary? The meaning of risk in multiplex case families with ankylosing spondylitis. | |
| 6 | Development of a checklist for patients with axial spondyloarthritis and psoriatic arthritis in daily practice: ONLY TOOLS project. | |
| Reason for exclusion: Not qualitative. (n=9) | | |
| 1 | A Benchmarking Study Evaluating Axial Spondyloarthritis Burden in Spain and Other European Countries. Results from the Spanish Atlas and the European Map of Axial Spondyloarthritis (EMAS) Studies. | |
| 2 | The information needs of people living with ankylosing spondylitis: a questionnaire survey. | |
| 3 | Patient Concerns and Perceptions Regarding Biologic Therapies in Ankylosing Spondylitis: Insights From a Large-Scale Survey of Social Media Platforms. | |
| 4 | Physical Activity in Ankylosing Spondylitis: evaluation and analysis of an eHealth tool. | |
| 5 | Delayed diagnosis is linked to worse outcomes and unfavourable treatment responses in patients with axial spondyloarthritis. | |
| 6 | Maintained activity in ankylosing spondylitis patients treated with TNFi and/or NSAID for at least 12 weeks: a cross-sectional study in Brazil. | |
| 7 | Reliability and Validity of Turkish Version of Short Form of the Social Role Participation Questionnaire in Patients With Ankylosing Spondylitis. | |
| 8 | Using Self-Reported Patient Experiences to Understand Patient Burden: Learnings from Digital Patient Communities in Ankylosing Spondylitis. | |
| 9 | Quality of Life and Related Factors in Patients With Ankylosing Spondylitis: A Cross-Sectional Study Using 36-Item Short Form Survey (SF-36). | |
| Reason for exclusion: Reviews (n=5) | | |
| 1 | Patient-reported outcomes in European spondyloarthritis patients: a systematic review of the literature. | |
| 2 | Physical activity and attitudes and perceptions towards physical activity in patients with spondyloarthritis: A systematic review. | |
| 3 | Inmune-mediated inflammatory rheumatic diseases in transgender people: A scoping review. | |
| 4 | Patient-perceived health service needs in inflammatory arthritis: A systematic scoping review. | |
| 5 | Exploring the emotional impact of axial Spondyloarthritis: a systematic review and thematic synthesis of qualitative studies and a review of social media. | |
| Reason for exclusion: Abstract only (n=3) | | |
| 1 | Patients' views on the management of ankylosing spondylitis: A qualitative study. | |
| 2 | Patients' expectations and needs with regard to rheumatology nursing care: results of multi-centre focus group interviews. | |
| 3 | Utilization Of An Informational Needs Assessment To Develop An Education Program For Patients With Ankylosing Spondylitis (AS) and Related Axial Spondyloarthritis (SpA). | |
