# Supplementary material for: Experiences and perceptions of patients with ankylosing spondylitis: A systematic review and meta-synthesis of qualitative studies
Source: PLoS One. 2024 Oct 17;19(10):e0311798. doi: 10.1371/journal.pone.0311798 (PMC11486380; doi:10.1371/journal.pone.0311798)
Supplement: S2 Table — (DOCX) [file pone.0311798.s004.docx]

| **Title** | **Extract themes** | **Data extractors** | **Date of data extraction** |
| --- | --- | --- | --- |
| 1 | Two themes were identified:  (1) physical and social limitations.  (2) emotional problems. | Nvivo | April 10-12, 2024 |
| 2 | Two themes were identified:  (1) activities of daily living.  (2) fatigue. |  |  |
| 3 | Three themes were identified:  (1) pervasive fatigue.  (2) current limitations (of self-management).  (3) a new direction (for future interventions). |  |  |
| 4 | Three themes were identified:  (1) patterns and causes of fatigue.  (2) consequences of fatigue.  (3) management of fatigue. |  |  |
| 5 | Four themes were identified:  (1) approaching a diagnosis.  (2) ill in a social context.  (3) challenged as a man.  (4) the importance of remaining physically well. |  |  |
| 6 | Two themes were identified:  (1) comprehensible, manageable, life strain-related tiredness.  (2) unfamiliar and unmanageable illness-related fatigue. |  |  |
| 7 | Three themes were identified:  (1) ordinary life condition.  (2) slowed-down life condition.  (3) disrupted life condition. |  |  |
| 8 | Three themes were identified:  (1) daily living and psychological reactions.  (2)a difficult diagnosis.  (3) working life and identity. |  |  |
| 9 | Three themes were identified:  (1) always with pain.  (2) the percevied limitation.  (3) fearing the unknown future. |  |  |
| 10 | Four themes were identified:  (1) impact of disease symptoms.  (2) limitations in life planning.  (3) experience of loneliness and isolation.  (4) multiple strategies for coping with disease. |  |  |
| 11 | Eleven themes were identified:  perception of abnormal somatic symptoms prior to diagnosis; tortuous experience of seeking medical treatment; prominent perception of adverse emotions after diagnosis; multiple ways of seeking information and support for the disease; perceived benefits of treatment; overly optimistic prognosis; ignoring the risk of progression of the disease during the period of recovery; skepticism about the controllability of the treatment; active or passive acceptance of the disease; declining level of social participation; desire for understanding and support. |  |  |

All data extracted from the primary research.

| **Title** | **Extract data** | **Data extractors** | **Date of data extraction** |
| --- | --- | --- | --- |
| 1 | “I never want to remember the really painful periods I have had. How can it be described?... I cannot find words to describe this situation. Just think: you cannot even put on your pants. I have never felt so helpless before.”  “My sex life has been very affected. Because of the very severe pain, I cannot have sex. I cannot adapt myself to sex because of the pain I feel. In fact, to lie down in bed, even for a very short time, increases my pain.”  “Going to the toilet was my biggest difficulty. You cannot sit on the toilet. If you can sit, you cannot stand up. This is a very bad situation. When it comes to cleaning yourself, you cannot bend or turn because of the pain.”  “In the future, there will be more pain affecting my life. For that reason, while my health is better, I should get married as soon as possible and have a child.”  “I do not think of a second pregnancy. My first pregnancy was a very difficult period. During and after my pregnancy, I had too much pain. I could not bend to pick up and nurse my baby. I do not want any more babies.”  “When you have pain, you do not think of your social life. You might want to think about it, but you cannot…In fact, I do not want to be with anybody during periods of pain.”  “Very difficult. For three months I could not get out of my house. I stopped going to the university...I was playing on the football team of my school, and now there is no football in my life.” | Nvivo | April 13, 2024 |
| 2 | “Fatigue and tiredness is something that I struggle with on a day-to-day basis. And in speaking with other rheumatologists in the past, it’s something that can be underemphasized probably. So, I love that it’s called out as one of the first questions there.” |  | April 13, 2024 |
| 3 | “I was taking indomethacin for the pain but it caused the bowel to flare up and I was hospitalised with that….so I try and manage without drugs if I can…but of course you have to if the pain is very bad.”  “I can’t remember ever having a good night’s sleep. I wake up constantly every couple of hours through pain, I never sleep through, so I am constantly tired.”  “a burning sensation with your eyes when you are really tired.”  “It would be lovely to swim in a warm pool but the prices these places are charging.” |  | April 13, 2024 |
| 4 | “It works, and the benefit is that I sleep better at night.”  “‘...I said, I can’t eat it, I feel ill’, and he said, ‘what’s wrong?’, and I said, ‘I have no energy, it’s all gone, it makes me feel ill.’”  “Because I’m tired most of the time, I can’t be bothered putting on make-up or doing my hair.”  “I have to write it down because if I don’t write it down it will get to three days later and I won’t have remembered it at all.” |  | April 13, 2024 |
| 5 | “Even today, I feel bad about getting paid for just staying home. I don’t contribute to anything. And it’s because I was raised to believe a man gets up in the morning to be the breadwinner for his family.”  “One or two have dropped out because they couldn’t really accept that you just couldn’t go out with them every weekend.”  “…it is difficult for my partner to accept that I cannot manage usual tasks.”  The men spoke about patient associations negatively, although the majority of them had memberships. They did not take advantage of their offers, and they expressed no need to: ‘…sit in a circle and hear about someone else’s pain.’ |  | April 14, 2024 |
| 6 | “It’s no wonder I’m so tired—I sleep so badly! And on top of everything it takes a lot of energy to explain the situation to the people at the job center who don’t understand the sort of jobs I can do. Now I hope things will fall into place soon so I can use my energy to live the way I want to.” |  | April 14, 2024 |
| 7 | “It’s not just bad, it’s so bad I can’t walk. It happened once at an airport where I had to use a wheelchair because I couldn’t stand up. It’s quite true, I couldn’t stand up—my legs, or my leg, it gave way when I stood on it.”  “I get noticeably more tired. It’s like the feeling that I can’t do anything. I’m a person who wants to do so much, I want to, I want to—but I don’t manage to.”  “I now manage a shop that sells clothes, shoes, handbags and things. Generally, I’m very pleased with this. It’s a fine job. But it wasn’t what I intended to do. Because when I’d finished studying and got a job and worked very hard like young academics do, I had to go on sick leave. I had inflammation in my hip joints and back and everywhere. I was on sick leave for three months.”  “I became a real patient—I must have been hospitalized about 70 or 80 times. I lost many of the friends I used to have. Yes, I did. First I lost contact with them for several years. And then you were very ill and things so you weren’t the same when you came back.” |  | April 14, 2024 |
| 8 | The physicians could not explain Janus’ pain attacks. It was not until a family member suggested that it could be AS that he had a magnetic resonance imaging (MRI) scan, was referred to a rheumatology department and soon after was diagnosed with AS – one year after the first episode of pain attacks.  Despite the daily pain and a hard, physically demanding job, Mikkel managed to do this job. When his pain grew worse, he eventually went to a chiropractor to obtain pain relief. The chiropractor referred him for an MRI of the spine. Shortly afterwards, Mikkel was diagnosed with AS – 13 years after symptom onset.  “I am frustrated that I have no stamina, that I’m such a weakling. That I can’t just suck it up and then stay at work, and that I have to go home early.”  “Well, I was unemployed and became depressed. It wasn’t serious, but my mood was like I wasn’t sad or anything, but I quickly became angry and upset, and if I talked to my family or something, I would snap all the time, and that wasn’t nice. I was sweating a lot and my heart pounded when I was going to sleep, so I couldn’t sleep and all that kind of stuff. So I was given antidepressants to take, which wasn’t nice – but that’s how it is.”  “What about the depression? I had mild to moderate depression, said my doctor. Not true, said the psychologist. It is moderate to severe depression that you have, and I actually think that you need some pills for it. I was already taking 16 pills daily.”  “the days when my back hurts - what can I actually do then? I can’t do anything."  “I am frustrated that I have no stamina, that I’m such a weakling. That I can’t just suck it up and then stay at work, and that I have to go home early.”  “…the days when my back hurts – what can I actually do then? I can’t do anything – Idefinitely can’t have a full-time job, so you can say that a lot of things are still unresolved.”  “I definitely can’t have a full-time job, so you can say that a lot of things are still unresolved.”  “…that I was retaining water in my hips, just like pregnant women. Then I was told that it was because I drank too much and partied too much-that it was because of that that I retained water in my hips.” |  | April 15, 2024 |
| 9 | “The highest level of pain is often in the mornings. I am not able to do my own responsibilities if I do not take my drugs as if living a normal life depends on medicines.”  “For this disease, my nerves are on edge and with a chronic pain in my life, my tolerance is less than I used to be and sometimes, I feel like crying.”  “I do not want to go out with my friends. I become more sensitive when they put their hand on my joints and bones.” |  | April 15, 2024 |
| 10 | “It also hurt before, but it wasn't so serious, so I didn't pay attention to it. Now the attack is quite severe. Only if there is no pain for one or two days a week, I think of coming to the hospital again.”  “It also hurt before, but it wasn't so serious, so I didn't pay attention to it. Now the attack is quite severe. Only if there is no pain for one or two days a week, I think of coming to the hospital again.”  “My wife and I were planning to have a second child, but then we found out about this disease, and I think I've heard that it has something to do with genetics, so I'd better forget about it.”  “To work as a customer service in a small county, although the salary is not high, the work is relatively loose and the body can bear it. During this period of time, the illness relapsed again. I have no choice but to quit.”  “I walked with a limp, so they (my friends) called me 'cripple' and stopped taking me to basketball games, which over time led to alienating myself from them.” |  | April 15, 2024 |
| 11 | “The orthopedic (doctor) told me to see the pain department, the pain department (doctor) told me to see the orthopedic department, and then the Chinese medicine department, and none of them could see what was wrong with me.”  “The town doctor recommended minimally invasive surgery at an orthopedic clinic, and then the knee still hurt, and it took 7 or 8 years of visits before it was diagnosed.”  “Some people on the internet say it's better to use oral medication, others say it's better (to use) biologics, and I don't know which (claim) to believe.”  “I'm only in my thirties, I can't find a wife, I can't find a job, do I have to rely on my parents?”  “The doctor said it's a hereditary disease. I'm afraid it'll affect the next generation.”  “How could a living, breathing person get this disease? I wanted to jump from the hospital.”  “When it's hard to go on a break, my coworkers might think I'm being hypocritical and uncomfortable when I'm not doing anything every day.”  “I hope the people around me can understand this disease, some things are not that I'm lazy and don't want to do, but I just can't do them.” |  | April 16, 2024 |

**The title represented by the serial number**

1: Living with pain in ankylosing spondylitis: a qualitative study.

2: A Novel Qualitative Study Assessing Patient-Reported Outcome Measures Among People Living with Psoriatic Arthritis or Ankylosing Spondylitis.

3: Patient perspectives of managing fatigue in Ankylosing Spondylitis, and views on potential interventions: a qualitative study.

4: Fatigue in ankylosing spondylitis: causes, consequences and self-management.

5: Men's experiences of living with ankylosing spondylitis: a qualitative study.

6: Life strain-related tiredness and illness-related fatigue in individuals with ankylosing spondylitis.

7: Living with a fluctuating illness of ankylosing spondylitis: a qualitative study.

8: A Difficult Diagnosis: A Qualitative Study of the Daily Lives of Young Men Diagnosed with Ankylosing Spondylitis.

9: The New Perceptions on Life of Iranian Patients with Ankylosing Spondylitis: A Qualitative Study.

10: Qualitative study on disease perception and experience of young and middle-aged patients with ankylosing spondylitis.

11: Qualitative study on the characteristics of disease perception in different stages of young patients with ankylosing spondylitis.
