# Supplementary material for: Experiences and perceptions of patients with ankylosing spondylitis: A systematic review and meta-synthesis of qualitative studies
Source: PLoS One. 2024 Oct 17;19(10):e0311798. doi: 10.1371/journal.pone.0311798 (PMC11486380; doi:10.1371/journal.pone.0311798)
Supplement: Attachment — Submitted filename: Reponse to Reviewers.docx [file pone.0311798.s006.docx]

Dear Editor and Reviewer,

Thank you very much for giving me this opportunity to revise our manuscript entitled “Experiences and perceptions of patients with ankylosing spondylitis: A systematic review and meta-synthesis of qualitative studies (PONE-D-24-24187R1)” again. According to the review comments, this revision is mainly for the abstract content in the system, and the manuscript content does not need to be modified.

Looking forward to hearing from you soon.

Yours Sincerely,

Yu Li

**Reponse to Editor’s Comments:**

**1. Please ensure that your decision is justified on PLOS ONE’s publication criteria and not, for example, on novelty or perceived impact.**

**Reponse:** Thank you for your valuable feedback. Through examination, our manuscript meets the criteria of plos one.

**2. For Lab, Study and Registered Report Protocols: These article types are not expected to include results but may include pilot data.**

**Reponse:** Thank you for your excellent suggestion. Our manuscript type is a systematic review and meta-synthesis, which contains the results of the study but does not require pilot data.

**Reponse to Reviewer 1’s Comments:**

**1. The phrase 'Intensive spinal colitis' still remains in the main abstract of the article which needs to be changed.**

**Reponse:** Thank you very much for your careful review. In the last revision, we have changed the phrase “intensive spinal colitis” in the manuscript to “ankylosing spondylitis”. However, due to an oversight, we did not check that the term “intensive spinal colitis” was not replaced in the summary of the system. In this submission, we have amended “intensive spinal colitis” to “ankylosing spondylitis” in the system.
